# Study on the Mechanism and Experiment of Styrene Butadiene Rubber Reinforcement by Spent Fluid Catalytic Cracking Catalyst

**DOI:** 10.3390/polym15041000

**Published:** 2023-02-17

**Authors:** Tilun Shan, Huiguang Bian, Donglin Zhu, Kongshuo Wang, Chuansheng Wang, Xiaolong Tian

**Affiliations:** 1 National Engineering Laboratory of Advanced Tire Equipment and Key Materials, Qingdao University of Science and Technology, Qingdao 266061, China; 2 Shandong Key Laboratory of Advanced Manufacturing of Polymer Materials, Qingdao 266061, China; 3 College of Electromechanical Engineering, Qingdao University of Science and Technology, Qingdao 266061, China

**Keywords:** spent FCC catalyst, physical modification, SBR composites, reinforcement mechanism

## Abstract

Spent Fluid Catalytic Cracking (FCC) Catalyst is a major waste in the field of the petroleum processing field, with a large output and serious pollution. The treatment cost of these waste catalysts is high, and how to achieve their efficient reuse has become a key topic of research at home and abroad. To this end, this paper conducted a mechanistic and experimental study on the replacement of some carbon blacks by spent FCC catalysts for the preparation of rubber products and explored the synergistic reinforcing effect of spent catalysts and carbon blacks, in order to extend the reuse methods of spent catalysts and reduce the pollution caused by them to the environment. The experimental results demonstrated that the filler dispersion and distribution in the compound are more uniform after replacing the carbon black with modified spent FCC catalysts. The crosslinking density of rubber increases, the Payne effect is decreased, and the dynamic mechanical properties and aging resistance are improved. When the number of replacement parts reached 15, the comprehensive performance of the rubber composites remained the same as that of the control group. In this paper, the spent FCC catalysts modified by the physical method instead of the carbon-black-filled SBR can not only improve the performance of rubber products, but also can provide basic technical and theoretical support to realize the recycling of spent FCC catalysts and reduce the environmental pressure. The feasibility of preparing rubber composites by spent catalysts is also verified.

## 1. Introduction

With the increase in car ownership, the demand for tires is growing rapidly every year [1,2]. Rubber composite is widely used in the tire industry for its excellent comprehensive performance and processing performance [3,4]. In order to meet the requirements of tire use, rubber compounds need to be prepared with a variety of reinforcing fillers to improve their performance [5,6]. In the process of tire preparation, the amount of filler accounts for more than 1/3 of the amount of tire rubber formula, and is becoming one of the most important materials in the tire rubber composition [7,8]. Compared with the high economic cost and serious environmental pollution caused by traditional fillers (carbon black), the “green tire” has become the main direction of automotive tire design and development [9,10,11,12].

Fluid Catalytic Cracking (FCC) is one of the important refinery processing technologies and has a pivotal position in the petroleum refining industry [13,14,15]. In the process of the petroleum catalytic cracking, the amount of the FCC catalyst is very large, and a large number of spent catalysts are also produced, which is called spent FCC catalysts in the industry [16]. This part of the spent FCC catalysts cannot meet the catalytic cracking requirements of petroleum; however, these catalysts still have high activity, and the residual activity of spent FCC catalysts can be used again in other fields. After our extensive preliminary research, we found that waste FCC catalysts applied to the waste tire pyrolysis process can significantly improve the quality of pyrolysis oil and meet the requirements of waste tire pyrolysis [17,18,19]. Therefore, the regeneration, recycling, and harmless application of spent catalysts is a key research direction in the future [20,21].

The FCC catalyst is a kind of porous, microsphere granular solid acid catalyst, which is made of active components (Y and ZSM-5 molecular sieve), matrix (kaolin), and binder (silica, alumina, etc.) by spray drying [22,23]. Kaolin, silica, and other substances are the main additives in rubber. Agustini S et al. [24] used kaolin as a filler for natural rubber to prepare solid tires for scooters. The vulcanization properties, mechanical properties, and thermal properties of the rubber compound were investigated. The experimental results demonstrated that the amount of kaolin had a great influence on the maximum torque, scorch time, optimum curing time, and mechanical properties of the vulcanized rubber. The thermogravimetric analysis demonstrated that the thermal stability of the rubber was influenced by the amount of the kaolin-filling fraction. Tan J et al. [25] ground anthracite coal to replace carbon-black-filled styrene–butadiene rubber (SBR) to prepare composites of styrene–butadiene rubber (SBR) and modified anthracite coal (MA). The experimental results demonstrate that the anthracite flakes can be well dispersed in the rubber matrix, providing good reinforcement properties. In addition, the low content of carbon black or silica composite fillers further promoted the dispersion of coal particles in the rubber, which effectively enhanced the mechanical reinforcing properties of coal particles and the thermal stability of rubber composites. Wang Z et al. [26] investigated the possibility of illite as an alternative natural rubber (NR) filler. The experimental results demonstrated that illite treated with cetyltrimethylammonium bromide (CTAB) could enhance the crosslinking density and dispersion of illite-NR, and the mechanical properties and wear resistance of illite/NR composites could be improved. Phuhiangpa N et al. [27] investigated the effect of the nano calcium carbonate (NCC) and micron calcium carbonate (MCC) on natural rubber composites. The experimental results demonstrate that two kinds of fillers (MCC and NCC) and filled rubber composites showed the same trend, but the effect of the small particle size in NCC on the composite properties was more pronounced and could be better used to adjust the rubber product characteristics and processing properties.

To extend the reuse methods of spent catalysts and reduce the pollution caused by them to the environment, this paper conducted a mechanistic and experimental study on the replacement of some carbon blacks by spent FCC catalysts for the preparation of rubber products, and the feasibility of the application of the spent catalysts to prepare rubber composite was also explored. The use of the spent FCC catalyst as a filler to replace part of the carbon black for rubber composite preparation can not only realize the recycling of waste rubber products, but also reduce the environmental pollution caused by the improper treatment of the spent catalyst. The feasibility of preparing rubber composites by spent catalysts is also verified.

## 2. Experiment

### 2.1. Experimental Scheme

To explore the influence of the number of replacement parts of spent FCC catalysts and the vulcanization system on the properties of rubber composites, the experimental formulations used in this study are shown in Table 1.

The purpose of the 1^#^–4^#^ in Table 1 is to study the influence of replacing different ratios of carbon black on the mechanical properties of the rubber composites before the modification of the spent FCC catalyst. The purpose of the 5^#^–7^#^ is to study the influence of particle size and pore size variation on the mechanical properties of rubber composites after the modification of spent FCC catalysts. The purpose of the 8^#^–10^#^ is to study the influence of adding the S, accelerator NS, and silane coupling agent Si69 on the reinforcement performance of the filler with the increase in the replacement amount of the modified spent FCC catalyst.

### 2.2. Experimental Process

#### 2.2.1. Spent FCC Catalyst Modification

**Figure 1:**
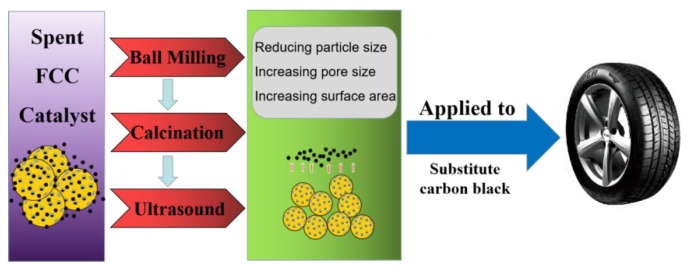
Flow chart of spent FCC catalyst modification.

First, the spent FCC Catalyst was ball milled (all-round planetary ball mill, QM-QX4, Nanjing Nanda Instruments Co., Ltd., Nanjing, China) at 180 r/min for 2 h. Then, the spent FCC catalyst was calcined (tube furnace, MFLGKD 405-12, Shanghai Muffle Furnace Technology Instruments Co., Ltd., Shanghai, China) at 600 °C for 3 h. Subsequently, the spent FCC catalyst and deionized water were mixed 1:5 for ultrasonic (ultrasonic disperser, VCY-1500, Shanghai Yanyong Ultrasonic Equipment Co., Ltd., Shanghai, China) treatment for 1 h, with continuous stirring during the ultrasonic process. Finally, the spent FCC catalyst was filtered and dried (electric blast drying oven, DHG-9240A, Shanghai Yiheng Scientific Instrument Co., Ltd., Shanghai, China) to complete the preparation of the spent FCC catalyst. The modification process of the spent FCC catalyst was roughly shown in Figure 1.

#### 2.2.2. Preparation of Rubber Composites

First, the butadiene rubber was plasticized into thin pieces of about 4mm in the open mixing machine (open mixing machine, X (S) K-160, Shanghai Rubber Machinery Factory, Shanghai, China) for later cutting and compacting. Subsequently, rubber, fillers (carbon black and spent FCC catalyst), and small materials were added to the mixer (laboratory small mixer, 0.3 L, Harbin Harper Electric Technology Co., Ltd., Harbin, China) to complete the preliminary preparation of rubber products. Then, the S and accelerator NS were added to the open mixing machine to complete the preparation of the compounded rubber. Finally, the mixed rubber pieces after standing for 12 h were used for the initial testing and vulcanization.

The vulcanization time T90 of each group of blends is measured by a rotorless sulfurometer (rotorless vulcanization instrument, M-2000-AN, high-speed rail detection instrument (Dongguan) Co., Ltd., Dongguan, China). Using a flat vulcanizing machine (flat vulcanizing machine, XLD-400X400X2, Qingdao Yilang Rubber Equipment Co., Ltd., Qingdao, China) for vulcanization, the vulcanization conditions are 150 °C × 1.3 T90.

### 2.3. Characterization

The Payne effect of the compound was tested by a rubber processing analyzer (Rubber Processing Analyzer, RPA2000, Alpha Technologies, Inc., Akron, OH, USA). The frequency was 1Hz, the strain range was between 0.28% and 40%, and the temperature was 60 °C.

The Mooney viscosity of the compound was tested by the Mooney viscometer (Mooney viscometer, PremierMV, Alpha Technology Co., Ltd., Akron, OH, USA) according to the standard ISO 289-1: 1994.

Vulcanization properties of the compound were tested by a rotorless rheometer according to the standard ISO 6502:1991, and the test temperature was 150 °C.

The hardness test of rubber was carried out using a Shore hardness tester (Shore hardness tester, LX-A, Shanghai Liuling Instrument Factory, Shanghai, China) according to the standard ISO 7619-1:2004. The rubber rebound rate was tested using a rubber rebound tester (rubber rebound tester, DIN-53512, Dongguan Songjiao Testing Instruments Co., Ltd., Dongguan, China) according to the standard ISO 4662-1986. The abrasion was tested by the DIN roller abrasion tester (DIN roller abrasion tester, GT-2012-D, Taichung Gao Tai Testing Machine Co., Ltd., Taiwan, China) according to the standard ISO 4649-2002. Tensile tearing properties were tested using the tensile testing machine (Tensile Testing Machine, AI-7000-MGD, Gautech Testing Instruments (Dongguan) Co., Ltd.) according to the standard of ISO 37-2005 and ISO 34-1:2004.

The dynamic mechanical properties of vulcanized rubber were tested by a dynamic thermo-mechanical analyzer (dynamic thermo-mechanical analyzer, EPLEXOR 150N, GABO, Ahlden, Germany). The dynamic strain was 0.25%, the static strain was 7%, the heating rate was 2 °C/min, the temperature range was −65~65 °C, and the frequency was 10 Hz.

The aging experiment of rubber was tested by using a hot air aging box (hot air aging box, GT-7017-M, High-Tech Testing Instruments Co., Ltd., Taiwan, China) according to the standard ISO 188-1998.

## 3. Results and Discussion

### 3.1. Analysis of Physical–Chemical Characteristics of Spent FCC Catalysts

**Figure 2:**
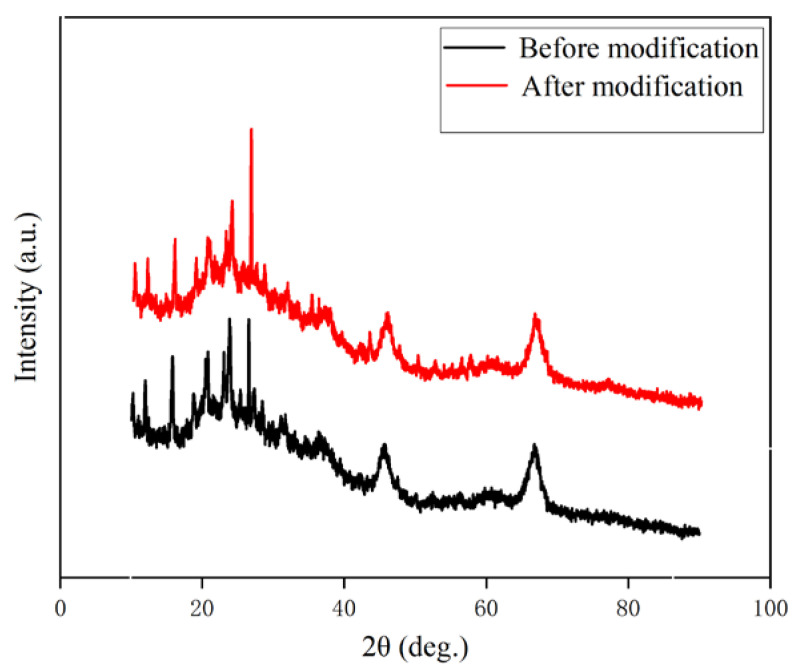
XRD spectrum of spent FCC catalyst.

The characterization results of XRD spectra of the spent FCC catalyst before and after modification are shown in Figure 2.

The crystal plane spacing of the spent FCC catalyst before and after the modification has not changed much in Figure 2. There was no new diffraction peak, indicating that the spent FCC catalyst did not change its phase composition after the modification [28,29].

**Figure 3:**
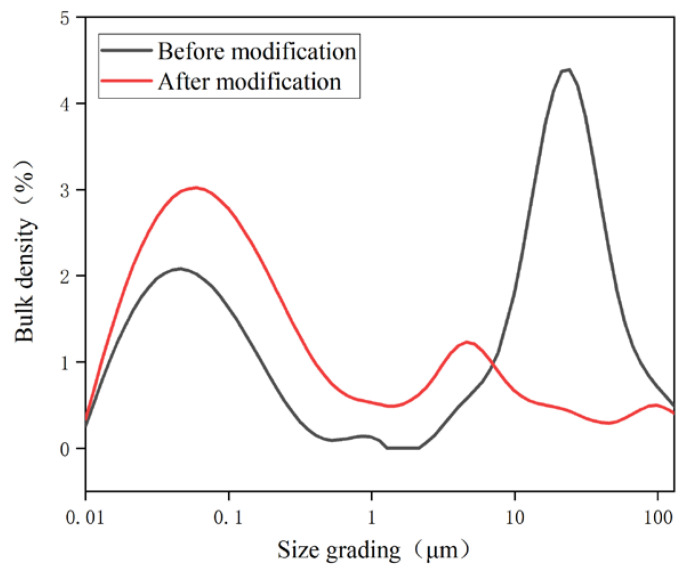
Particle size distribution of spent FCC catalyst.

The volume change and distribution of the particle size of the spent FCC catalyst before and after the modification are shown in Table 2 and Figure 3.

The particle size of the spent FCC catalyst decreased significantly after a modification from Table 2 and Figure 3. The average particle size of the spent FCC catalyst decreased from 12.8 μm to 0.163 μm, which may be due to the serious agglomeration phenomenon before the modification of the spent FCC catalyst. During the ball milling process, the collision between the ball, the cylinder wall, and the spent FCC catalyst or the spent FCC catalyst self-grinded with each other, which contributed to the particle size reduction.

The changes of the specific surface area of the spent FCC catalyst before and after the modification are shown in Table 3.

**Figure 4:**
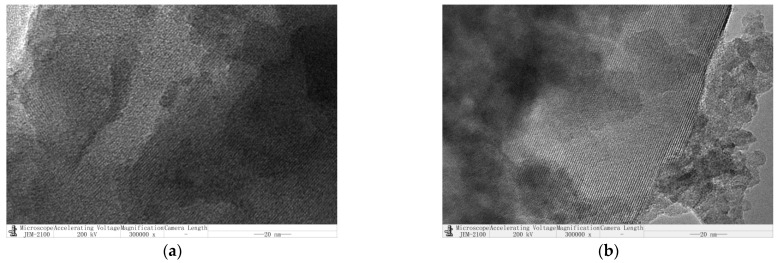
Changes in pore morphology of spent FCC catalysts before and after modification. (**a**) Before modification; (**b**) after modification.

The changes of the pore size morphology before and after the modification of spent FCC catalysts are shown in Figure 4.

From Table 3 and Figure 4, it can be observed that the specific surface area and pore volume of the spent FCC catalyst increase after the modification. Because the ball milling reduces the catalyst particle size; the combination of calcination and sonication reduces the adsorbed material on the catalyst (on the surface and inside the pores), thus increasing the specific surface area and pore volume [30].

**Figure 5:**
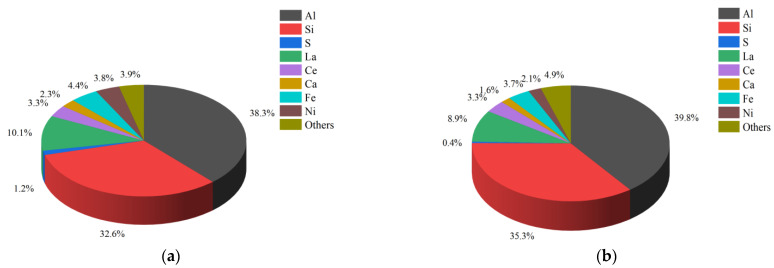
Relative proportions of main elements. (**a**) Before modification; (**b**) after modification.

The relative percentages of major elements before and after the modification of the spent FCC catalysts are shown in Figure 5.

It can be observed from Figure 5 that the elements contained in the spent FCC catalyst before the modification mainly include aluminum (Al), silicon (Si), lanthanum (La), etc. The relative content of Si and Al elements increased after the modification of the spent FCC catalyst, while the content of the cerium (Ce) remained basically unchanged and the relative content of the other elements decreased. This may be due to the volatilization of coke and other substances adsorbed from crude oil during the high-temperature calcination process, which caused the elemental content of the spent FCC catalyst to change.

### 3.2. Processing Performance Analysis of Rubber Composites

The rubber vulcanization characteristics of the different proportions of carbon black replaced by the spent FCC catalyst before the modification are shown in Table 4.

The rubber vulcanization characteristics of modified spent FCC catalyst replacing carbon black with different proportions are shown in Table 5.

From Table 4, it can be observed that with the increase in the number of parts of carbon black replaced by the spent FCC catalyst, the Mooney viscosity of the rubber compound demonstrated a decreasing trend, indicating that the addition of the spent FCC catalyst improved the plasticity and processability of the compound [31]. The value of MH-ML is considered to be positively related to the crosslinking density of the rubber [32,33]. The higher the value, the higher the crosslinking density. According to the data in the table, the MH-ML value decreases as the number of parts increases, which is related to the weak reinforcement of the spent FCC catalyst and is consistent with the trend of the Menny viscosity. T10 is generally considered to be related to the rubber scorch time; the larger the value, the higher the processing safety, and vice versa [34]. It can be observed from the data that the processing safety decreases as the number of replacement parts of the spent FCC catalyst increases. The elemental analysis demonstrates that the spent FCC catalyst contains elements such as S, which leads to early local cross-linking during the mixing process, therefore resulting in scorching. T90 is the positive vulcanization time. From the data, it can be observed that as the number of replacement parts increases, the positive vulcanization time becomes longer, which leads to an increase in vulcanization time and a decrease in economic efficiency [35].

As can be observed from Table 5, the reinforcement of the modified spent FCC catalyst is greatly improved, and it can be used as a reinforcing filler to replace a part of the carbon black for the rubber filler. From the MH-ML difference, T10, and T90 in the data of columns 5^#^–7^#^, it can be observed that the crosslink density is significantly increased compared with that before the modification. There is a slight increase in T10, and the processing safety is improved. There is a significant decrease in the positive vulcanization time of T90. This is due to the decrease in the particle size, the increase in the specific surface area and pore volume, and the decrease in impurities of the modified spent FCC catalyst, which leads to the enhancement of interfacial bonding with the polymer and the improvement of performance [36]. From the data in columns 8^#^–10^#^, it can be observed that S, accelerator NS, and the silane coupling agent Si69 increased equiproportionally with the increase in the spent FCC catalyst replacement. The MH-ML difference and T10 and T90 parameters are greatly improved; thus, the performance is even more excellent.

### 3.3. Rubber Composite Payne Effect Analysis

**Figure 6:**
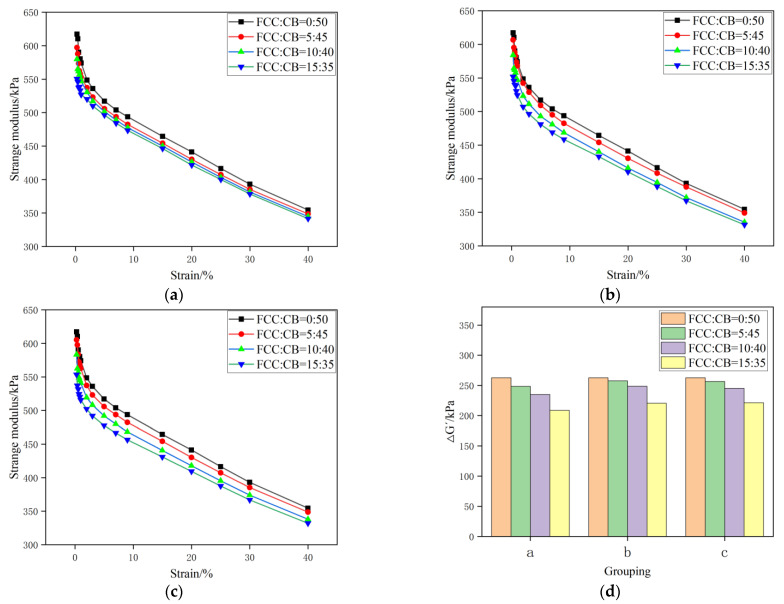
Storage modulus change diagram of rubber composite (abscissa a, b, and c in (**d**) correspond to three storage modulus change diagrams: a, b, and c, respectively). (**a**) Formulation 1^#^–4^#^; (**b**) formulation 1^#^, 5^#^–7^#^; (**c**) formulation 1^#^, 8^#^-1; (**d**) change of storage modulus.

The change of the storage modulus of rubber composites with different parts of the carbon black replaced by the spent FCC catalyst is shown in Figure 6.

Figure 6 is the storage modulus and strain curve of rubber composites under the spent FCC catalyst/carbon black. With the increase in strain, the storage modulus of filler–filled rubber composites decreases, which is called the Payne effect [37,38]. ΔG′ represents the degree of the network structure of the filler, and ΔG′ is the difference between the storage modulus at a 40% strain and the storage modulus at a 0.28% strain. The smaller ΔG′ indicates that the Payne effect is weak and the filler has a better dispersion in the rubber matrix. From Figure 6d, it can be observed that with the increase in the number of carbon black parts replaced by the spent FCC catalyst, the ΔG′ of the compounded rubber shows a decreasing trend. This is mainly because the spent FCC catalyst can form more filler–rubber network structures with rubber, with less filler agglomeration, and exhibit a low modulus in torsion tests; thus, the Payne effect is weakened.

### 3.4. Analysis of Physical and Mechanical Properties of Rubber Composites

The function of fillers in rubber products is mostly to improve the mechanical properties such as hardness, tensile strength, and elongation at the break of rubber composites. The mechanical properties corresponding to different experimental formulations in Table 1 are shown in Table 6 (Table 1, formulation 1^#^–4^#^) and Table 7 (Table 1, formulation 5^#^–10^#^).

As shown in Table 6, the mechanical properties of the rubber material filled with the spent FCC catalyst directly instead of the carbon black rubber were poor. The tensile strength, tear strength, and abrasion properties decreased significantly as the number of replacement parts increased.

From Table 7 (formulation 5^#^–7^#^), it can be observed that the hardness and tensile stress of the rubber composites demonstrated a decreasing trend as the number of replacement parts of the spent FCC catalyst increased. This may be due to the combination of the spent FCC catalyst with the rubber molecular chain being weaker compared to the carbon black, resulting in the decrease in the hardness and tensile stress of the rubber composites.

From Table 7 (formulation 8^#^–10^#^), it can be observed that with the increase in the number of replacement carbon black parts after the modification of the spent FCC catalyst, the fixed tensile strength, tensile strength, and tear strength of the rubber composites were enhanced after the addition of S, accelerator NS, and the silane coupling agent Si69 in an equal proportion, so that the properties of the three groups of the rubber composites remained basically the same as the control group (formulation 1^#^). This is mainly because with the increase in the accelerator NS, S, and silane coupling agent Si69, the interfacial bonding between silica and rubber molecular chains in the spent FCC catalyst is enhanced. At the same time, the increase in the accelerator NS and S improves the degree of vulcanization and increases the crosslinking density; this shows the characteristics of a high modulus and high elongation.

The wear resistance of rubber composites after replacing the carbon black with the spent FCC catalyst was slightly lower than that of the control group. This may be due to the particle size of the spent FCC catalyst being larger than that of the carbon black (N660 particle size 49–60 nm), and the combination of the spent FCC catalyst and rubber molecular chains is weaker compared to the carbon black, which leads to the easy fall off of the spent FCC catalyst during the process of abrasion. After the spent FCC catalyst falls off, the surface of the rubber composite is defective, resulting in more rubber particles being worn off and therefore with increased wear. The spent FCC catalysts contain reinforcing substances such as SiO_2_. With the increase in the amount of the accelerator NS, S and silane coupling agent Si69, the interfacial combination of silica in the spent FCC catalyst and rubber molecular chain is enhanced, and the spent FCC catalyst does not easily fall off during the wear process; thus, the wear consumption is reduced.

### 3.5. Microscopic Morphology

**Figure 7:**
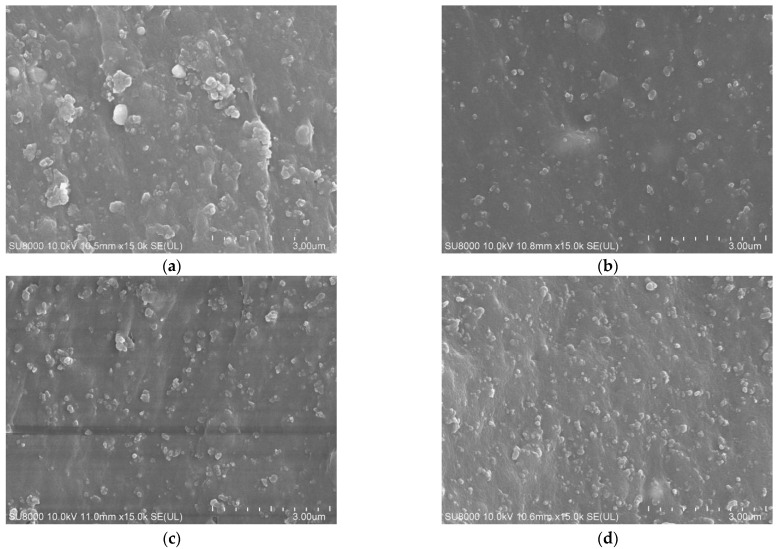
Scanning electron micrograph of rubber composites prepared by replacing part of carbon black with spent FCC catalyst. (**a**) 1^#^; (**b**) 5^#^; (**c**) 6^#^; (**d**) 7^#^.

The microscopic morphologies corresponding to the rubber composites prepared by formulations 1^#^, 5^#^, 6^#^, and 7^#^ are shown in Figure 7.

**Figure 8:**
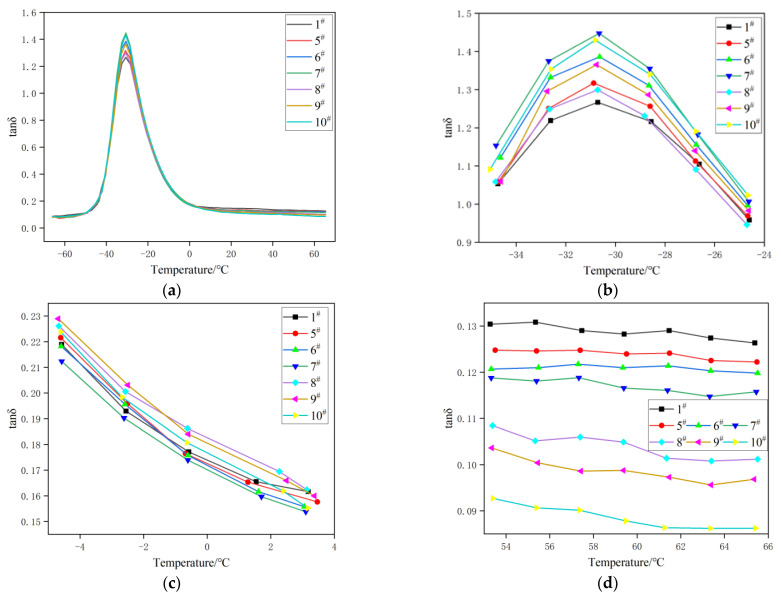
Dynamic mechanical properties of rubber composites.(**a**) tanδ-T Curve of the composites; (**b**) is magnified graphs of curves around −30 °C; (**c**) is magnified graphs of curves around 0 °C; (**d**) is magnified graphs of curves around 60 °C.

As can be observed from Figure 7, the modified spent FCC catalyst, instead of the carbon black filler in the rubber, has a better dispersion and no more obvious agglomeration phenomenon. When 15 phr of the modified spent FCC catalyst was used to replace the equal mass of the carbon black, the dispersion and distribution of the filler in the rubber was optimal, which was consistent with the Payne effect results shown in Figure 8.

### 3.6. Dynamic Properties Analysis of Rubber Composites

Figure 8 shows the change curve of the loss factor (tanδ) with a temperature for rubber composites at −65~65 °C [39,40]. Usually, the higher the peak value of tanδ, the better the dispersion of the filler. The tanδ at 0 °C represents the wet skid resistance of the tire, and the larger the value, the better the wet skid resistance. The tanδ at 60 °C represents the rolling resistance of the tire, and the smaller the value, the lower the rolling resistance. The peak value of tanδ from high to low is 7^#^ ≈ 10^#^ > 6^#^ ≈ 9^#^ > 5^#^ ≈ 8^#^ > 1^#^, which is basically consistent with the characterization results in Figure 6, indicating that the replacement of the carbon black by the spent FCC catalyst for rubber filling can improve the dispersion of the filler in the rubber matrix. At 0 °C, the curves of formulation 5^#^–7^#^ are lower than those of the control group (formulation 1^#^), indicating that the wet skid resistance of the tires decreases after replacing the carbon black with the spent FCC catalyst. The corresponding curves of formulation 8^#^–10^#^ are higher than those of the control group (formulation 1^#^). With the increase in the number of replacement parts of spent FCC catalysts, the equal proportional addition of S, accelerator NS and the silane coupling agent Si69 improved the anti-slip properties of the tires. Mao C et al. [41] believed that there is an inevitable relationship between the surface roughness of the sample and the wet skid resistance. The rougher the surface, the higher the coefficient of the wet friction and the better the wet skid resistance. Therefore, the wet skid resistance of the rubber polymer in this experiment may be related to the particle size of the spent FCC catalyst and the interfacial bonding strength of the filler and the rubber. At 60 °C, the corresponding curves of formulations 5^#^–10^#^ are lower than those of formulation 1^#^, while the corresponding curves of formulations 8^#^–10^#^ continue to show a downward trend and are lower than those of formulations 5^#^–7^#^, indicating that with the increase in replacement parts of the spent FCC catalyst, the addition of S, accelerator NS and the silane coupling agent Si69 in equal proportion could reduce the rolling resistance of the tire.

### 3.7. Aging Properties’ Analysis

The aging properties of the vulcanized rubber composites (formulations 1^#^–10^#^) were tested after aging at 100 °C for 24 h. The aging tensile properties are shown in Table 8.

As shown in Table 8, the aging property change rates of the elongation at the break, and the tensile strength and tensile product of the rubber composites prepared by replacing the carbon black with spent FCC catalysts (formulations 5^#^–7^#^), are lower than those of the control group (formulation 1^#^), indicating that the aging resistance is better than that of the control group. With the increase in the number of replacement parts of the spent FCC catalyst, the aging property change rate of the composites was reduced again by adding S, accelerator NS and the silane coupling agent Si69 (formulation 8^#^–10^#^) in equal proportion; the anti-aging property is improved again and is significantly better than that of the control group. This is mainly because the spent FCC catalyst is porous material and can adsorb some of the vulcanization system and other rubber additives to become the carrier of the slow-release agent. During the aging process, the S absorbed in the pore size is released and re-involved in the vulcanization reaction. At the same time, the silane coupling agent Si69 contains four elemental sulfur, which will also be re-involved in the vulcanization reaction during the aging process to enhance the anti-aging properties of rubber.

### 3.8. Synergistic Reinforcement Mechanism of Spent FCC Catalyst and Carbon Black for SBR

The spent FCC catalyst is uniformly and disorderly dispersed in the SBR matrix. During the mixing process of the filler (spent FCC catalyst) and rubber, rubber molecular chains are adsorbed on the surface of the filler to form bound rubber; meanwhile, some rubber molecular chains enter the voids and pores of the filler to form a small amount of inclusion rubber (retention rubber). The interaction between the spent FCC catalyst and rubber mainly includes the physical adsorption and chemical combination, which can form a close filler–polymer network structure with rubber, so as to improve the basic physical properties of rubber products. When the rubber products are stretched by an external force, the existence of the bound rubber is conducive to prolonging the crack expansion path so that the rubber can withstand a greater external force, and the existence of the inclusion rubber is conducive to limiting the displacement of the molecular chains around the filler, thereby improving the mechanical properties of the rubber.

## 4. Conclusions

In this paper, we explore the spent FCC catalysts to replace different parts of the carbon black for the rubber product preparation to realize the resource utilization and diversified applications of spent FCC catalysts. The use of the spent FCC catalyst as a filler to replace part of the carbon black for the rubber composite preparation can not only realize the recycling of waste rubber products, but also reduce the environmental pollution caused by the improper treatment of the spent catalyst.

The spent FCC catalyst as a filler to replace carbon black in the rubber has good dispersibility, which is conducive to the improvement of the comprehensive performance of rubber products (stretching, stretching, rebound, tearing, etc.). The Payne effect is decreased by about 3–16%, and the rolling resistance is obviously reduced. The spent FCC catalyst is a porous material, which can be used as a carrier of the slow release agent. In the aging process, S in the pore size of the spent FCC catalyst will be released again to participate in the vulcanization reaction and improve the anti-aging properties of rubber products.

## Figures and Tables

**Table 1:** Experimental formulation.

Samples	1^#^	2^#^	3^#^	4^#^	5^#^	6^#^	7^#^	8^#^	9^#^	10^#^	Manufacturer
SBR1500	100	100	100	100	100	100	100	100	100	100	PetroChina Dushanzi Petrochemical Company, Karamay, China
Zinc oxide	3	3	3	3	3	3	3	3	3	3	Shijiazhuang Yunpo Chemical Technology Co., Ltd., Shijiazhuang, China
Accelerator NS	1	1	1	1	1	1	1	1.07	1.13	1.2	Shandong Shangshun Chemical Co., Ltd., Weifang, China
Sulphur (S)	1.75	1.75	1.75	1.75	1.75	1.75	1.75	1.87	1.98	2.1	Chaoyang Tianming Industry & Trade Co., Ltd., Beijing, China
Silane coupling agent Si69	\	\	\	\	\	\	\	0.25	0.5	0.75	Shandong Xiya Chemical Co., Ltd., Linyi, China
Carbon black N660	50	45	40	35	45	40	35	45	40	35	Shanghai Cabot Chemical Co., Ltd., Shanghai, China
Spent fcc catalyst	\	5	10	15	\	\	\	\	\	\	Sinopec Jinan Oil Refinery, Jinan, China
Modified spent FCC catalyst	\	\	\	\	5	10	15	5	10	15	Sinopec Jinan Oil Refinery, Jinan, China

Note: The unit of component dosage in the table is g.

**Table 2:** Changes in particle size and volume of spent FCC catalyst before and after modification.

	Before Modification	After Modification	Reduce Proportion (%)
Dv (10)/μm	0.0306	0.0259	15.36
Dv (50)/μm	12.8	0.163	98.73
Dv (90)/μm	55.0	18.7	66.0

**Table 3:** Brunauer–Emmett–Teller (BET) characterization results.

	Specific Surface Area (m^2^/g)	Substrate Surface (m^2^/g)	Micropore Surface Area (m^2^/g)	Total Pore Volume (mL/g)	Micropore Volume (mL/g)
Before modification	75	17	58	0.1564	0.0104
After modification	89	29	60	0.1884	0.0152

**Table 4:** Vulcanization characteristics of rubber composites with spent FCC catalyst before modification.

	1^#^	2^#^	3^#^	4^#^
Mooney viscosity/MU	70.90	70.14	69.27	68.77
ML/(N·m)	2.13	2.06	1.87	2.19
MH/(N·m)	20.27	12.75	9.96	9.85
MH-ML/(N·m)	18.14	10.69	8.09	7.66
T10/min	11.92	10.07	10.2	8.87
T90/min	29.53	36.83	44.11	46.00
T100/min	56.33	59.59	59.72	59.93

Note: 1^#^, 2^#^, 3^#^, respectively corresponding to the experimental group in Table 1.

**Table 5:** Vulcanization characteristics of rubber composites after modification with spent FCC catalyst.

	5^#^	6^#^	7^#^	8^#^	9^#^	10^#^
Mooney viscosity/MU	70.68	69.13	68.95	70.31	69.27	68.46
ML/(N·m)	1.94	1.92	1.84	2.15	2.08	1.99
MH/(N·m)	18.43	18.2	16.12	22.08	21.70	21.29
MH-ML/(N·m)	16.53	16.28	14.28	19.93	19.62	19.30
T10/min	10.8	12.05	12.22	10.22	11.06	11.27
T90/min	31.47	36.03	38.50	26.69	27.70	28.33
T100/min	59.18	59.87	59.95	48.75	52.91	53.00

Note: 5^#^, 6^#^, 7^#^, 8^#^, 9^#^, 10^#^ respectively corresponding to the experimental group in Table 1.

**Table 6:** Mechanical properties of vulcanized rubber.

	1^#^	2^#^	3^#^	4^#^
Hardness/Shore A	60.0	48.5	45.5	45.0
10% tensile stress/MPa	0.54	0.56	0.55	0.54
100% tensile stress/MPa	2.74	1.67	1.36	1.18
300% tensile stress/MPa	12.82	6.57	4.25	2.55
Tensile strength/MPa	19.61	18.22	13.62	8.48
Elongation at break/%	454.85	657.80	693.20	752.91
Tensile product	8919.61	11,985.12	9441.38	6384.68
Tearing strength/N	77.78	54.39	50.41	39.54
Specific gravity/g·cm^−3^	1.142	1.149	1.156	1.163
DIN abrasion/cm^3^	0.108	0.137	0.149	0.166
Rebound rate/%	60.3	60.9	61.4	61.7

Note: 1^#^, 2^#^, 3^#^, respectively corresponding to the experimental group in Table 1.

**Table 7:** Mechanical properties of vulcanized rubber.

	5^#^	6^#^	7^#^	8^#^	9^#^	10^#^
Hardness/Shore A	58.0	57.5	55.0	62.0	61.5	61.0
10% tensile stress/MPa	0.60	0.56	0.53	0.62	0.57	0.56
100% tensile stress/MPa	2.54	2.37	2.04	3.16	3.01	3.00
300% tensile stress/MPa	11.03	9.67	7.50	13.12	13.10	12.88
Tensile strength/MPa	19.46	19.35	19.01	20.04	19.95	19.79
Elongation at break/%	478.22	505.41	580.31	441.34	443.60	443.87
Tensile product	9306.16	9830.22	11,031.69	8846.31	8848.27	8784.10
Tearing strength/N	73.68	71.44	68.37	87.13	83.25	81.46
Specific gravity/g·cm^−3^	1.146	1.152	1.162	1.147	1.153	1.161
DIN abrasion/cm^3^	0.128	0.129	0.131	0.109	0.119	0.124
Rebound rate/%	62.0	62.5	63.1	64.00	65.50	66.70

Note: 5^#^, 6^#^, 7^#^, 8^#^, 9^#^, 10^#^ respectively corresponding to the experimental group in Table 1.

**Table 8:** Aging tensile properties of rubber composites.

Test Items		1^#^	5^#^	6^#^	7^#^	8^#^	9^#^	10^#^
Elongation at break/%	Before aging	454.85	478.22	505.41	580.31	441.34	443.60	443.87
	After aging	270.49	311.26	331.82	385.11	295.08	297.68	298.86
Aging property change rate/%		40.53	34.91	34.35	33.64	33.14	32.89	32.67
Tensile strength/ MPa	Before aging	19.61	19.46	19.35	19.01	20.04	19.85	19.79
	After aging	16.82	18.24	17.43	16.82	19.06	18.49	18.28
Aging property change rate/%		14.23	6.27	9.92	11.52	4.89	6.85	8.59
Tensile product	Before aging	8919.61	9306.16	9779.68	11,031.69	8844.45	8805.46	8784.19
	After aging	4550.26	5677.38	5783.62	6477.55	5624.22	5504.10	5463.16
Aging property change rate/%		48.99	38.99	40.86	41.28	36.41	37.49	37.81
Aging coefficient		0.51	0.61	0.59	0.59	0.64	0.63	0.62

## Data Availability

The data supporting the findings of this study are available within the article.
